# Economic impact of a care bundle to prevent surgical site infection after craniotomy: a cost-analysis study

**DOI:** 10.1186/s13756-021-01016-4

**Published:** 2021-10-13

**Authors:** Emilio Jiménez-Martínez, Guillermo Cuervo, Jordi Carratalà, Ana Hornero, Pilar Ciercoles, Andreu Gabarrós, Carmen Cabellos, Ivan Pelegrin, Maria Angeles Domínguez-Luzón, Jordi Càmara, Ramon Moreno-Fuentes, Jordi Adamuz, Miquel Pujol

**Affiliations:** 1 Infectious Diseases Department, Hospital Universitari de Bellvitge - Institut d’Investigació Biomèdica de Bellvitge (IDIBELL), Feixa Llarga s/n, 08907 L’Hospitalet de Llobregat, Barcelona, Spain; 2 Neurosurgery Department, Hospital Universitari de Bellvitge - Institut d’Investigació Biomèdica de Bellvitge (IDIBELL), Barcelona, Spain; 3 Infectious Diseases Department, Parc de Salut Mar, Barcelona, Spain; 4 Microbiology Department, Hospital Universitari de Bellvitge - Institut d’Investigació Biomèdica de Bellvitge (IDIBELL), Barcelona, Spain; 5 Finance Department, Hospital Universitari de Bellvitge - Institut d’Investigació Biomèdica de Bellvitge (IDIBELL), Barcelona, Spain; 6 Nursing Information Systems Department Support, Hospital Universitari de Bellvitge - Institut d’Investigació Biomèdica de Bellvitge (IDIBELL), Barcelona, Spain; 7 University of Barcelona, Barcelona, Spain; 8 Spanish Network for Research in Infectious Diseases (REIPI), Instituto Carlos III, Madrid, Spain; 9 Research Network for Respiratory Diseases (CIBERES), ISCIII, Madrid, Spain

**Keywords:** Surgical site infection, Craniotomy, Bundle, Prevention, Economic impact

## Abstract

**Background:**

Surgical site infections after craniotomy (SSI-CRAN) significantly impact patient outcomes and healthcare costs by increasing length of stay and readmission and reoperation rates. However, to our knowledge, no study has yet analysed the economic impact of a surgical care bundle for preventing SSI-CRAN. The aim is to analyse the hospital cost saving after implementation of a care bundle for the prevention of SSI-CRAN.

**Methods:**

A retrospective cost-analysis was performed, considering two periods: pre-care bundle (2013–2015) and care bundle (2016–2017). A bottom-up approach was used to calculate the costs associated with infection in patients who developed a SSI-CRAN in comparison to those who did not, in both periods and on a patient-by-patient basis. The derived cost of SSI-CRAN was calculated considering: (1) cost of the antibiotic treatment, (2) cost of length of stay in the neurosurgery ward within the 1-year follow up period, (3) cost of the re-intervention, and (4) cost of the implant for cranial reconstruction, when necessary.

**Results:**

A total of 595 patients were included in the pre-care bundle period and 422 in the care bundle period. Mean cost of a craniotomy procedure was approximately €8000, rising to €24,000 in the case of SSI-CRAN. Mean yearly hospital costs fell by €502,857 in the care bundle period (€714,886 vs. €212,029). Extra costs between periods were mainly due to increased length of hospital stay (€573,555.3 vs. €183,958.9; difference: €389,596.4), followed by the cost of implant for cranial reconstruction (€69,803.4 vs. €9,936; difference: €59,867.4). Overall, implementation of the care bundle saved the hospital €500,844.3/year.

**Conclusion:**

The implementation of a care bundle for SSI-CRAN had a significant economic impact. Hospitals should consider the deployment of this multimodal preventive strategy to reduce their SSI-CRAN rates, and also their costs.

**Supplementary Information:**

The online version contains supplementary material available at 10.1186/s13756-021-01016-4.

## Background

Surgical site infections are among the most preventable health-care-associated infections. They entail a great economic burden for the health-care systems due to their associated morbidity and mortality and the loss in patients’ quality of life [1]. The treatment of any surgical site infection increases the cost of the intervention by roughly €17,000, rising to €80,000 if the treatment involves the replacement of a prosthesis or if the infection is due to a multidrug-resistant microorganism [2, 3].

Thus, surgical site infections contribute to excess healthcare utilization and increase costs. In a scenario of growing pressure to maximize economic resources, institutions need to optimize costs and improve the quality of the healthcare provided. A better understanding of the risk factors for surgical site infection and the possible preventive measures is therefore required, as well as an appraisal of its economic impact [4]. Due to time-dependent nature of neurosurgical infections, its economic burden is often difficult to estimate.

In the field of neurosurgery, research on the prevention of surgical site infection after craniotomy (SSI-CRAN) has focused on how care bundles might help to bring down infection rates [5–8]. Although data are scarce, the attributable cost of SSI-CRAN has been determined [9, 10]; however, the economic impact of the implementation of a care bundle to prevent SSI-CRAN has not been calculated.

The aim of this study was to analyse the economic impact on hospital costs of the implementation of a care bundle for the prevention of SSI-CRAN.

## Methods

### Study design, setting and patients

A retrospective cost-analysis study was performed at Bellvitge University Hospital, a 700-bed university hospital for adults in Barcelona, Spain. All consecutive adult patients without any active infection undergoing a clean open craniotomy, according to the criteria of the Centers for Disease Control and Prevention (CDC), were prospectively followed up and included in the study [11]. In December 2015, after noting the high rates of SSI-CRANs, a multidisciplinary team was created to implement a preventive care bundle. The bundle measures took into account the risk factors for, and causative agents of, SSI-CRANs identified in a previous study [12], as well as the most up-to-date clinical practice guidelines regarding measures for preventing surgical site infections [13–16]. The interventions and the effectiveness of the care bundle were evaluated in a previous study [8], which reported a reduction in SSI-CRAN from 15.3 to 3.5%. To assess the economic impact of the implementation of the measure, the study was divided into two periods: “pre-care bundle” (from 1 January 2013 to 31 December 2015) and “care bundle” (from 1 January 2016 to 31 December 2017). All patients who underwent craniotomy up to December 31th 2015 received pre, intra, and post-IQ care measures corresponding to the “pre-care bundle” period. In this way, it was avoided that the overlap between periods affects the interpretation of the results and the cost analysis after the application of the bundle.

### Data collection and SSI-CRAN surveillance

Data collection and follow-up during the study period were performed by members of the infection control team, who had received specific training in surveillance methodology so as to ensure the collection of homogeneous and accurate data. All patients were prospectively recruited through hospital surveillance under the surgical site infection program. Patients undergoing craniotomy were prospectively followed from the day of intervention up to 1 year post-surgery to determine the incidence of SSI-CRAN. Active mandatory post-discharge surveillance included the following: (a) review of electronic clinical charts (primary and secondary care); (b) assessment of readmissions; (c) evaluation of emergency visits; and (d) review of microbiological and radiological data during the period of post-discharge surveillance.

### Variables, data source and study outcomes

The primary study endpoint was to determine the difference in cost between non-SSI-CRAN and SSI-CRAN patients 1 year post-surgery. Basic demographic data were recorded, along with the following information on patient comorbidities and surgical procedures: the Charlson comorbidity score [17]; the American Society of Anaesthesiologists (ASA) classification, type of surgery (elective or emergency), reason for surgery (intrinsic tumour, extrinsic tumour, epilepsy, vascular diseases, trauma or other), operative site (supratentorial, infratentorial or retromastoid), administration of antibiotic prophylaxis according to hospital guidelines, duration of surgery, use of intracranial pressure monitors, re-intervention, cerebrospinal fluid (CSF) leak and metal plates; characteristics of the infection using CDC classification and bacterial aetiology; and in-hospital outcome data (pre- and post-surgery in-hospital stay). SSI-CRAN secondary cost was calculated by considering the following healthcare components: (1) cost of the antibiotic treatment, (2) cost of length of stay in the neurosurgery ward during the 1-year follow up, (3) cost of the re-intervention, and (4) cost of the implant for cranial reconstruction, when necessary (Additional file 1).

First, to estimate the average cost, the characteristics of the patients who developed SSI-CRANs were compared with those who did not in both periods. Second, the two periods were compared to assess the economic impact on hospital healthcare costs.

### Definitions

In accordance with CDC criteria [11], a SSI-CRAN was defined as superficial incisional, deep incisional, or organ-space. The reason for surgery was defined by the underlying disease of the patient, which included: (a) an intrinsic tumour in the central nervous system parenchyma, (b) an extrinsic tumour in the structures of the central nervous system in the skull and meninges, (c) epilepsy, (d) vascular diseases, (e) traumatic injuries and (f) other.

Intravenous antibiotic prophylaxis was considered adequate when all the following criteria were met: antibiotics were administered according to the local protocol, the infusion ended within 60 min of the surgical incision, and perioperative re-dosing was performed according to duration if indicated. Thirty-day mortality was defined as death occurring due to any cause within 30 days of surgery.

The length of hospital stay was considered as the duration of hospitalization in the first admission, while the 1-year length of hospital stay was defined as the total number of days of hospitalization within the year following the neurosurgical intervention due to infections or other neurosurgical complications.

Days of antibiotic therapy were defined as the duration of either empirical or targeted treatment. The type and duration of antibiotic therapy were agreed by neurosurgeons and by the infectious disease consultant in accordance with the local protocol.

### Economic evaluation

A bottom-up approach to calculating costs associated with infection was used for patients who developed an SSI-CRAN and compared with those who did not, on a patient-by-patient basis. Costs were provided by the hospital’s finance department. Costs of length of hospital stay were calculated for all patients based on the current 2017 cost of one night’s stay in the neurosurgery ward. For each patient SSI-CRAN costs were calculated, including the following healthcare cost components considered independently: (1) 1-year length of hospital stay, (2) cost of re-intervention, (3) cost of implant, and (4) cost of antibiotic treatment. The components of healthcare costs were analysed separately to establish which of them had the greatest influence on the increase in expenditure.

Costs for patients with and without SSI-CRAN were calculated analysing the mean cost per craniotomy and the duration of hospitalization in the first admission and during the following year. Then, in patients with SSI-CRAN, the additional costs, such as cost of re-intervention, cost of implant, and cost of antibiotic treatment were added. The aim of this analysis was to calculate the mean cost of craniotomies and SSI-CRAN and also the global cost between periods.

Secondly, given that the number of interventions was not homogeneous between periods, the mean annual cost of interventions in patients with and without SSI-CRAN between periods was analysed. The aim of this analysis was to adjust healthcare spending for 1 year, so that the results could be compared with each other.

Finally, in order to show the favourable economic impact of the implementation of the care bundle, we compared the mean cost of bundle measures per year including: (1) systemic antibiotic prophylaxis: Cefuroxime 1.5 g (strong recommendation supported by high to moderate-quality evidence) [2], (2) topical antibiotic prophylaxis: Vancomycin powder 1 g in subgaleal space (unresolved issue) [18], and (3) sterile absorbent drape between periods (weak recommendation) [2], as well as the mean cost of SSI-CRAN per year between periods. Thus, the cost of the care bundle is reflected and, specifically, the saving the hospital obtained thanks to its implementation.

### Statistical methods

Quantitative variables are reported as medians and interquartile range (IQR); categorical variables are reported as absolute numbers and percentages. To identify significant differences between groups, the chi-square test or the Fisher’s exact test was used for categorical variables, and the Student *t* test or Mann–Whitney *U* test for continuous variables, as appropriate. The statistical analysis was performed with version 25.0 of the SPSS software package (SPSS, Chicago, IL). Statistical significance was established at α = 0.05, and all reported *p* values are two-tailed.

### Ethical issues

The study includes anonymised routine surveillance data. The need for informed consent and the information sheet was waived because the follow-up of patients undergoing craniotomy was included in our center’s surgical site infections surveillance program. The study was approved by the Clinical Research Ethics Committee of Bellvitge University Hospital (Reference number PR334/18).

## Results

### Baseline characteristics and outcomes

Overall, during the study period, 1017 patients undergoing a craniotomy were included. 595 in the pre-care bundle period and 422 in the care bundle period. Patients’ baseline and clinical characteristics in both periods are summarized in Table 1. The most significant differences between groups were: supratentorial surgical site (75.5% vs. 86%, *p* < 0.001) and inappropriate antibiotic prophylaxis (13.9% vs. 25.1%, *p* < 0.001), both higher in the care bundle period.

**Table 1:** Baseline characteristics and outcomes of patients undergoing craniotomy in the two study periods

	Pre-care bundle period (n = 595)		Care bundle period (n = 422)		*p* value
*Baseline characteristics*					
Male, n (%)	274	(54.6)	228	(45.4)	**0.013**
Median age, years (IQR)	54	(21)	55	(22)	0.143
Urgent surgery, n (%)	79	(13.3)	54	(12.8)	0.851
Reason for surgery					
Intrinsic tumour, n (%)	234	(39.3)	181	(42.9)	0.271
Extrinsic tumour, n (%)	123	(20.7)	105	(24.9)	0.127
Epilepsy, n (%)	23	(3.9)	8	(1.9)	0.095
Vascular, n (%)	152	(25.5)	100	(23.7)	0.508
Trauma, n (%)	17	(2.9)	20	(4.7)	0.127
Others, n (%)	46	(7.7)	8	(1.9)	< 0.001
Surgical site					
Supratentorial, n (%)	449	(75.5)	363	(86)	< 0.001
Infratentorial, n (%)	62	(10.4)	29	(6.9)	**0.05**
Retromastoid, n (%)	84	(14.1)	30	(7.1)	< 0.001
Duration of surgery: minutes, median (IQR)	243	(130)	260	(136.5)	0.08
CHARLSON score, median (IQR)	3	(2)	3	(2)	0.8
ASA > 2	223	(37.5)	127	(30.1)	0.08
Inappropriate antibiotic prophylaxis, n (%)	83	(13.9)	106	(25.1)	< 0.001
CSF leak, n (%)	11	(1.8)	3	(0.7)	0.173
ICP sensor, n (%)	36	(6.1)	23	(5.4)	0.786
Use of steroids/chemotherapy, n (%)	439	(73.8)	377	(89.3)	< 0.001
Mean hospital 1 year stay, median (IQR)	10	(19)	9.5	(16.2)	0.338
*Outcomes*					
Primary outcome					
SSI-CRAN^1^, n (%)	91	(15.3)	15	(3.5)	< 0.001
Occurrence of SSI-CRAN, days (IQR^2^)	21	(14–43)	19	(17.5–39.5)	0.06
Detection					
During hospital admission, n (%)	31	(34.1)	5	(33.3)	1
Post-discharge surveillance, n (%)	11	(12.1)	0		0.357
Readmission, n (%)	49	(53.8)	10	(66.7)	0.411
SSI-CRAN classification					
Superficial incisional, n (%)	8	(8.8)	2	(13.3)	0.777
Deep incisional, n (%)	16	(17.6)	3	(20)	0.730
Organ-space, n (%)	67	(73.6)	10	(66.7)	0.548
*Secondary SSI-CRAN Outcomes*	(n = 91)		(n = 15)		
1-year mortality, n (%)	2	(2.2)	0	(0)	1
Re-intervention, n (%)	72	(79.1)	13	(86.7)	0.730
Number of re-interventions, median (IQR)	1.4	(1.1)	1.4	(0.9)	0.891
Implant for cranial reconstruction	27	(29.7)	3	(20)	0.548
Days of antibiotic therapy, median (IQR)	37.8	(29.9)	35	(22.2)	0.764
Cumulative days of antibiotic therapy, days	3438		525		< 0.001
MDR^3^ microorganism infection, n (%)	16	(17.6)	3	(20)	0.730

*ASA* American Society of Anesthesiologists, *CSF* cerebrospinal fluid, *ICP* intracranial pressure, *IQR* interquartile range, *MDR* multidrug-resistant, *SSI-CRAN* surgical site infection after craniotomy

As Table 1 shows, the incidence of SSI-CRANs was significantly lower in the care bundle period (15.3% vs. 3.5%; *p* < 0.001). No significant differences were found between groups regarding type of infection and time elapsed from surgery to infection. Cumulative days of antibiotic therapy for SSI-CRAN were higher in the pre-care bundle period (3438 vs. 525 days, *p* < 0.001).

### Cost analysis

Using the unit cost from Additional file 1, craniotomy and SSI-CRAN-attributable costs are summarized in Table 2. The mean cost of a craniotomy was roughly €8,000, (€8,030.8, 95% CI 7350.6–8710.9), and that of a SSI-CRAN was around €24,000 (€24,248.5, 95% CI 21,180.6–27,316.4). Evaluating healthcare components, mean length of stay within 1 year was 16 days (16.5 ± 23.5) for non-SSI-CRAN patients and 47 days (42.8 ± 31.5) in SSI-CRAN patients; the mean cost of a re-intervention was €989.4 ± 72.8, and that of an implant was €2,163 ± 405.4 in SSI-CRAN patients. The mean cost of antibiotic treatment was €1,366.6 ± 172.37.

**Table 2:** Cost attributable to SSI-CRAN

	Study period 2013–17 (n = 1017)								
	No SSI-CRAN (n = 911)	SSI-CRAN (n = 106)							
		Superficial incisional (n = 10)		Deep incisional (n = 19)		Organ-space (n = 77)		Total (n = 106)	
*1-year hospital stay*									
Mean days ± SE	16.5 ± 23.5	31.8 ± 40.6		27.1 ± 28.7		48.1 ± 29.5		42.8 ± 31.5	
Mean cost (€) ± SE	8,030.4 ± 346.5	14,827.9 ± 5,710.8		12,740.7 ± 2,930.4		22,088.1 ± 1.494.2		19,727.7 ± 1,358.3	
95% CI	7,350.3– 8,710.4	1,909.3–27,746.7		6,584.2–18,897.2		19,112.1–25,063.9		17,034.5–22,420.9	
Sub-total cost (€)	6,687,078	141,379.62		228,963.85		1,647,650.54		2,017,994	
*Cost of re-intervention (€)*									
Number of cases, n (%)	–	3	(30)	10	(52.6)	72	(93.5)	85	(80.2)
Mean re-intervention per patient ± SE	–	0.5 ± 0.3		0.6 ± 0.2		1.7 ± 0.1		1.4 ± 0.1	
Mean cost ± SE	–	414 ± 234.5		508.4 ± 138		1182.9 ± 79.5		989.4 ± 72.8	
95% CI	–	116.6–944.6		218.4–798.4		1,024–1,341.2		845–1133.8	
Sub-total cost (€)	–	3600		8400		79,200		91,200	
*Cost of implant (€)*									
Number of cases, n (%)	–	1	(10)	1	(5.2)	28	(36.4)	30	(28.3)
Mean cost ± SE	–	–		–		2712 ± 514.5		2163 ± 405.4	
95% CI	–	–		–		1687.4–3736.6		1359.2–2966.8	
Sub-total cost (€)	–	10,820		9635.5		208,826.8		229,282.42	
*Cost of antibiotic treatment (€)*									
Number of cases, n (%)	–	5	(50)	13	(68.4)	71	(92.2)	89	(83.9)
Mean cost ± SE	–	1097.8 ± 728.29		804.6 ± 258.7		1540.2 ± 207.1		1366.6 ± 172.37	
95% CI	–	549.7–2745.3		261.1–1348.2		1127.7–1952.7		1024.8–1708.4	
Sub-total cost (€)	–	10,977.7		15,287.7		118,594.4		144,859.8	
*Total cost (€)*									
Number of cases	911	10		19		77		106	
Mean cost ± SE	8,030.8 ± 346.5	17,424.8 ± 6,686.7		14,560.9 ± 3,564.1		27,525 ± 1,611.7		24,248.5 ± 1,547.2	
95% CI	7350.6–8710.9	2298.5–32,551.2		7073.1–22,048.7		24,315.2–30,735.1		21,180.6–27,316.4	
Total cost	7,315,668	172,567.3		276,207.1		2,116,941		2,568,716.1	

*CI* confidence interval, *SE* standard error, *SSI-CRAN* surgical site infection after craniotomy

Table 3 displays the mean cost per year for non-SSI-CRAN and SSI-CRAN patients in the two periods. Comparing patients with SSI-CRAN, the mean cost per year fell by €502,857 in the care bundle period (from €714,886 in the pre-care bundle period to €212,029). Breaking down this mean cost according to healthcare components, extra cost was mainly driven by length of hospital stay within 1 year, with a difference of €389,596.4 (€573,555.3 pre-care bundle vs. €183,958.9 in the care bundle period), followed by the cost of implant for cranial reconstruction (€69,803.4 vs. €9936, difference: €59,867.4).

**Table 3:** Cost per year for 1-year hospital stay, re-intervention, implant and antibiotic treatment for non-SSI-CRAN and SSI-CRAN between study periods

	Non-SSI-CRAN			SSI-CRAN		
	Pre-care bundle period	Care bundle period	Difference	Pre-care bundle period	Care bundle period	Difference
1-year hospital stay (€)	1,287,118.3	1,727,156.5	− 440,038.2	573,555.3	183,958.9	389,596.4
Re-intervention (€)	–	–	–	30,130	6300	23,830
Implant (€)	–	–	–	69,803.4	9936	59,867.4
Antibiotic treatment (€)	–	–	–	40,397.3	11,834	28,563.3
Total cost (€)	1,287,118.3	1,727,156.5	− 440,038.2	714,886	212,029	502,857

The costs of the implementation of the care bundle are summarized in Table 4. Although its implementation entailed an outlay of €3101.7/year (compared to €1089/year in the pre-care bundle period), the hospital saved €500,844.3/year by preventing SSI-CRAN episodes (€715,975/year pre-care bundle vs. €215,130.7/year in the care bundle period).

**Table 4:** Mean cost of preventive measures between periods per year and mean cost of SSI-CRAN between periods per year

	Cost/unit (€)	Cost/craniotomy (€)	Cost/year (€)
*Mean cost of preventive measures between periods*			
Pre-care bundle period^a^			
Systemic antibiotic prophylaxis: Cefuroxime 1.5 g	2.6	5.2	1029.6
Sterile impermeable drape	0.3	0.3	59.4
Total cost	2.9	5.5	1089
Care bundle period^a^			
Systemic antibiotic prophylaxis: cefuroxime 1.5 g	2.6	5.2	1097.2
Topical antibiotic prophylaxis: vancomycin 1 g	6.9	6.9	1455.9
Sterile absorbent drape	0.6	2.6	548.6
Total cost	10.1	14.7	3101.7

	SSI-CRAN cost/year	Measures cost/year	Total cost
*Mean cost of SSI-CRAN between periods*			
Pre-care bundle period	714,886	1089	715,975
Care bundle period	212,029	3101.7	215,130.7
Difference of cost between periods	–	–	500,844.3

^a^Common costs between periods are not included (ex: antiseptic soap)

## Discussion

This study of a large cohort of patients undergoing a craniotomy demonstrates the impact of a care bundle in the reduction of health-care costs attributed to SSI-CRAN. With an annual implementation cost of 3101.7€ per year for the care bundle, the health-care system saved 500,844.7€ by preventing SSI-CRAN. The SSI-CRAN cost is mainly driven by the increase in length of hospital stay within the year of follow-up and the cost of implant for cranial reconstruction.

Few studies have examined the costs related to SSI-CRAN [9, 10]. Previous reports present results similar to ours, with attributable costs of €8000 for craniotomy and €24,000 for SSI-CRAN. Moreover, the cost increased when the SSI-CRAN diagnosed was organ-space. This infection has potentially devastating consequences: it requires complex treatment that often involves the removal of the bone flap and the placement of an implant for cranial reconstruction, and long-term antibiotic therapy, which lengthens hospital stay and increases the readmission rate [10, 19]. We stress that most SSI-CRANs were detected in the post-discharge surveillance period, and require hospital readmission. Our findings concur with the results of other studies [19, 20] suggesting that the practice of limiting follow-up to 30 days or the first hospital admission would cause several cases to be missed. Active surveillance must continue for at least 1 year after surgery in order to carry out a time-dependent cost-analysis study, as suggested previously [21].

As demonstrated in our earlier study [8], the implementation of a specific care bundle was effective in preventing SSI-CRANs. Furthermore, the current cost-analysis shows that in the pre-care bundle period, SSI-CRAN entailed an economic burden of more than €700,000 per year. Given the significant reduction of SSI-CRAN achieved in the bundle period, the attributable cost per year of these infections was around €200,000, a reduction of more than €500,000. The positive economic impact for the health-care system of the implementation of care bundles is well documented [22–24], but to the best of our knowledge, this is the first report of a significant reduction in health-care thanks to the implementation of a care bundle for the prevention of SSI-CRAN.

To optimize the use of healthcare resources, it is crucial to have a detailed analysis of all the costs that an infection entails and of its overall economic impact. In the case of SSI-CRAN, regardless of the length of hospital stay, the implant for cranial reconstruction is the element that incurs the highest economic expense. These results are in agreement with other studies [25, 26] that have shown the cost differences between autogenous and custom patient-specific implants. The main target of surgical site infection prevention in this setting is to avoid cranial osteomyelitis and bone flap deterioration—a complication that is devastating for the patient in terms of their quality of life, and extremely costly for the health-care system. Moreover, addressing this complication requires the use of antibiotic treatment and multiple re-interventions [20]. Antibiotic treatment, the second most expensive element, not only raises health-care costs but may also increase bacterial resistance [27, 28]. Finally, the consequence of an infection is an increase in the 1-year hospital stay, on average more than 40 days in patients with an SSI-CRAN in our study. The economic impact of the implementation of the care bundle is evident in this regard, since it allowed a saving of almost €400,000 per year. To highlight, in our study, the economic expense related to 1-year hospital stay in non-infected patients was higher in the care bundle period, probably because in this period more patients underwent a craniotomy annually compared to the pre-care bundle period. (195 craniotomies/year in the pre-care bundle period vs. 203 craniotomies/year in the care bundle period).

Our study has several limitations that should be noted. Firstly, it was carried out at a single center; although the characteristics of the SSI-CRANs were similar to those observed in other hospitals, the frequency of infections and the impact of measures may vary between settings. Secondly, some cost components (total parenteral nutrition, treatment or readmission to other hospitals, laboratory analyses and radiological diagnostic tests) were not included. Despite these limitations, some major strengths of the present study should be highlighted. Firstly, it was carried out at a reference center serving over 1,500,000 people in southern Catalonia as part of a prospective SSI-CRAN surveillance program and included a large cohort of consecutive patients undergoing craniotomy. Secondly, in our previous study [8] this care bundle intervention has demonstrated its effectiveness in reducing the incidence of SSI-CRAN.

## Conclusion

The implementation of a care bundle for the prevention of SSI-CRAN had a positive economic impact for the health-care system, allowing considerable cost-saving. Hospitals should incorporate the surveillance of surgical infections in the area of neurosurgery and should implement strategies such as the bundle described here in order to reduce the burden of SSI, in terms of both patient suffering and healthcare costs.

## Supplementary Information

**Additional file 1**. Resources and units costs.

## Data Availability

Not applicable.

## Abbreviations

ASA: American Society of Anesthesiologists
CDC: Centers for Disease Control and Prevention
CI: Confidence interval
CSF: Cerebro spinal fluid
ICP: Intracranial pressure
IQR: Interquartile range
MDR: Multidrug-resistant
SE: Standard error
SSI-CRAN: Surgical site infection after craniotomy
