# Supplementary material for: Economic impact of a care bundle to prevent surgical site infection after craniotomy: a cost-analysis study
Source: Antimicrob Resist Infect Control. 2021 Oct 13;10:146. doi: 10.1186/s13756-021-01016-4 (PMC8513215; doi:10.1186/s13756-021-01016-4)
Supplement: Supplementary file 1 — Additional file 1. Resources and units costs. [file 13756_2021_1016_MOESM1_ESM.docx]

**Supplementary Material 1: *Resources and units costs***

| CRANIOTOMY PROCEDURE (COST PER INTERVENTION) | | | | |
| --- | --- | --- | --- | --- |
| TYPE OF PROCEDURE: | | | | COST (€) |
|  | *Exploratory craniotomy* | | | 382.6 |
|  | *Decompressive craniotomy secondary trauma* | | | 585.8 |
|  | *Decompressive craniotomy secondary severe trauma* | | | 817.8 |
|  | *Decompressive craniotomy secondary vascular disease* | | | 539.9 |
|  | *Extradural hematoma drainage* | | | 736.6 |
|  | *Others (ex: deep brain stimulation)* | | | 413.8 |
| BED-HOSPITAL COST | | | | |
| Neurosurgical ward (bed/day) | | | | 444.6 |
| IMPLANT FOR CRANIAL RECONSTRUCTION | | | | |
| TYPES OF IMPLANT: | | | | COST (€) |
|  | | *Computer designed cranioplasty implant* | | 9,000 - 10,000 |
|  | | *Surgical cement (methilmetacrilate)* | | 72.6 |
|  | | *Metal plates* | | 200 - 400 |
|  | | *Metal screws* | | 200 - 400 |
|  | | *Titanium mesh* | | 2,000 – 3,000 |
| ANTIBIOTIC TREATMENT USE IN SSI-CRAN (COST PER DAY) | | | | |
|  | ANTIBIOTIC | | DOSE | COST/ DAY € |
|  | *Amikacine* | | 15mg/kg/24h | 3.3 |
|  | *Amoxicillin/Clavulanic* | | 1g/8h | 2.8 |
|  | *Ampicillin* | | 1g/6h | 2.1 |
|  | *Aztreonam* | | 1g/8h | 28.5 |
|  | *Cefazollin* | | 2g/8h | 4.6 |
|  | *Ceftazidime* | | 2g/8h | 6.7 |
|  | *Ceftazidime/Avibactam* | | 2g/8h | 422.9 |
|  | *Ceftriaxone* | | 1g/24h | 4.9 |
|  | *Cefuroxime* | | 750mg/8h | 3.9 |
|  | *Ciprofloxacin* | | 200mg/12h | 16.7 |
|  | *Clindamycin* | | 600mg/6h | 6.1 |
|  | *Cloxacillin* | | 2g/4h | 7.8 |
|  | *Co-trymoxazole* | | 1600mg/8h | 11.2 |
|  | *Daptomycin* | | 8-10mg/kg/24h | 200.0 |
|  | *Levofloxacin* | | 500mg/24h | 13.5 |
|  | *Linezolid* | | 600mg/12h | 119.2 |
|  | *Meropenem* | | 2g/8h | 70.7 |
|  | *Penicillin G* | | 2MIU/4h | 8.1 |
|  | *Piperacillin/Tazobactam* | | 4g/8h | 21.0 |
|  | *Vancomicine* | | 1g/12h | 13.8 |
| *G: grams, Kg: kilograms, IU: International Units, Mg: Milligrams,* | | | | |
